# Utility of transmanubrial osteomuscular sparing approach and its modification in vascular surgery: a case series study of surgeries related to subclavian artery

**DOI:** 10.1186/s44215-024-00158-2

**Published:** 2024-06-20

**Authors:** Kota Itagaki, Shintaro Katahira, Katsuhiro Hosoyama, Yusuke Suzuki, Hiromichi Niikawa, Masayuki Otani, Ryuichi Taketomi, Koki Ito, Goro Takahashi, Kiichiro Kumagai, Yoshinori Okada, Yoshikatsu Saiki

**Affiliations:** 1 Division of Cardiovascular Surgery, Tohoku University Hospital, 1-1, Seiryo-machi, Aoba-ku, Sendai, Miyagi 980-8574 Japan; 2 Department of Thoracic Surgery, Tohoku University Hospital, Sendai, Japan

**Keywords:** Clavicle, Manubrium, Transmanubrial approach, Aneurysm, Subclavian artery, Injury, Iatrogenic trauma, Vascular surgery, Catheter

## Abstract

**Background:**

The operative field in subclavian vessel surgery is limited by thoracic inlet and outlet structures. Although endovascular therapy for the subclavian artery could be an option, open repair management is occasionally required in cases of large aneurysms, infectious vasculopathy, and trauma. The transmanubrial osteomuscular sparing approach, commonly used in thoracic surgery area to resect superior sulcus tumors, is a simple and safe procedure providing an excellent view of the operative field. Herein, we present three cases that underwent open repair of the subclavian artery using the transmanubrial osteomuscular sparing approach, and we also highlight the utility of the technique along with the procedural details.

**Case presentation:**

Case 1: A 54-year-old man presented with a true aneurysm of the proximal portion of the right subclavian artery. The aneurysm measured 50 × 80 mm and compressed the right lung and trachea. We performed an aneurysm resection and a right subclavian artery reconstruction via the transmanubrial osteomuscular sparing approach under cardiopulmonary bypass support.

Case 2: A 72-year-old man who presented with an abscess that formed around the left subclavian artery due to an unremoved guidewire during thoracic endovascular aortic repair for an aortic arch aneurysm in another hospital. After the antibiotics administration, debridement and axillary-axillary bypass were performed, and the guidewire was removed via a transmanubrial osteomuscular sparing approach with a use of cardiopulmonary bypass.

Case 3: A 60-year-old man presented with misplacement of an indwelling dialysis catheter inserted for acute renal failure and hyperkalemia. The catheter was placed through the right neck, but had penetrated the right internal jugular vein and was misplaced from the right subclavian artery into the proximal aortic arch. Emergently, we removed the catheter using the transmanubrial osteomuscular sparing approach.

**Conclusions:**

The transmanubrial osteomuscular sparing approach to the subclavian artery provides an excellent view and a wide surgical field, even in different pathological situations. This is a simple, safe, and highly useful procedure and could be the standard approach for subclavian artery surgeries.

**Supplementary Information:**

The online version contains supplementary material available at 10.1186/s44215-024-00158-2.

## Background

**Fig. 1:**
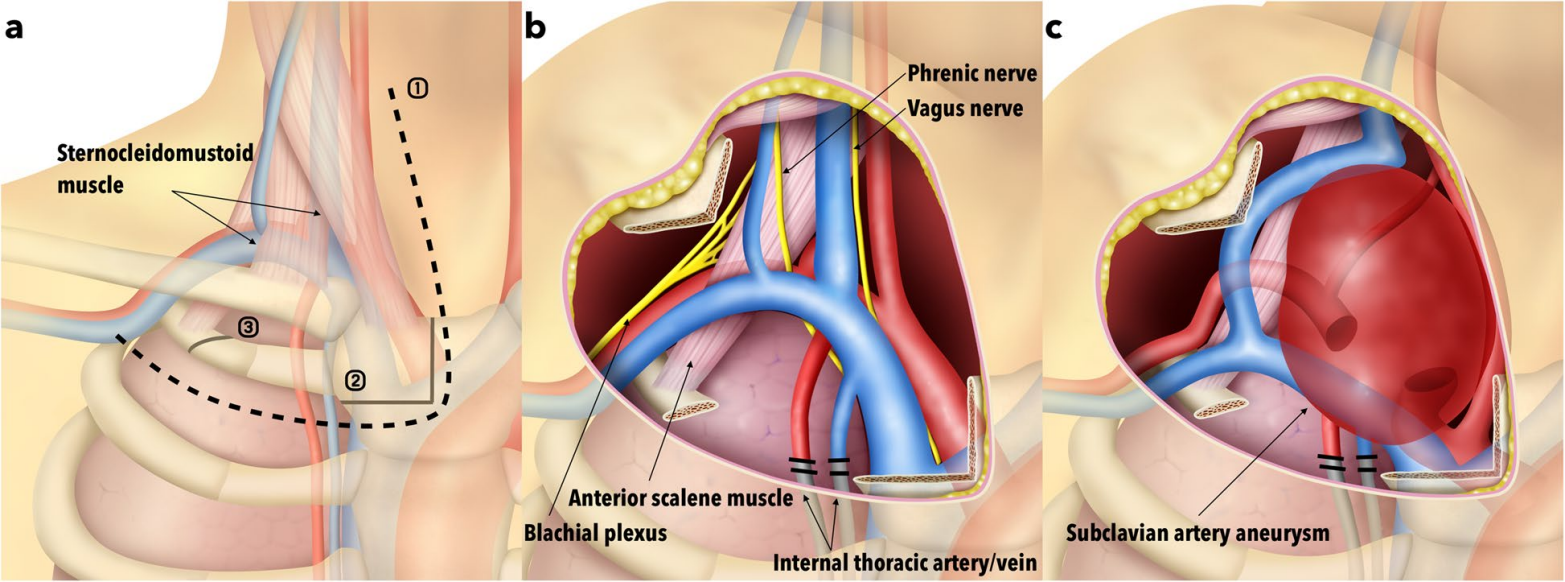
Schematic diagram of transmanubrial osteomuscular sparing approach in vascular surgery. **a** The figure shows an L-shaped incision across the manubrium with the upper line along the anterior margin of the sternocleidomastoid muscle and parallel to the first intercostal space (dotted line ①). The upper quarter of the manubrium is cut off (solid line ②) and the first rib is divided to lift the osteomuscular flap laterally (solid line ③). **b** Exposure through the transmanubrial osteomuscular sparing approach. The subclavian vein is exposed in a position anterior to the subclavian artery. In this surgical view, the proximal segment of the subclavian artery can be exposed even more easily. The internal thoracic artery and vein are inevitably divided during this procedure; however, the other structures are preserved. If necessary, the anterior scalene muscle can be resected, and the distal portion of the SCA can be revealed. **c** An example of schematic surgical view through the transmanubrial osteomuscular sparing approach applied in a patient developing a large subclavian artery aneurysm, as in the case 1. The approach can provide sufficient exposure of the entire aneurysm to facilitate dissection of surrounding structures and reconstruction of vital vessels with technical ease

Surgical approaches to the subclavian artery (SCA) could be complicated due to the surrounding components including the clavicle, first rib, lung, subclavian vein, and brachial plexus, restricting a view of the surgical field and working space. Although endovascular therapy could be an option in some cases [1–3], open surgeries are occasionally inevitable in conditions such as large aneurysms compressing vital organs, infectious vasculopathy, and trauma [2, 4, 5]. In 1997, Grunenwald et al. reported a case series of the transmanubrial osteomuscular sparing approach (TMA) for the resection of apical chest tumors. Since then, this approach has been widely performed in the thoracic surgery area [6], while this is somehow unfamiliar for vascular surgeons. We found that the TMA provided ideal exposure and a secure surgical view of the SCA (Fig. 1a, b, c). Herein, we report three cases who underwent open surgery for SCA lesions using TMA, and highlight the advantages of this approach.

## Case presentation

### Case 1

**Fig. 2:**
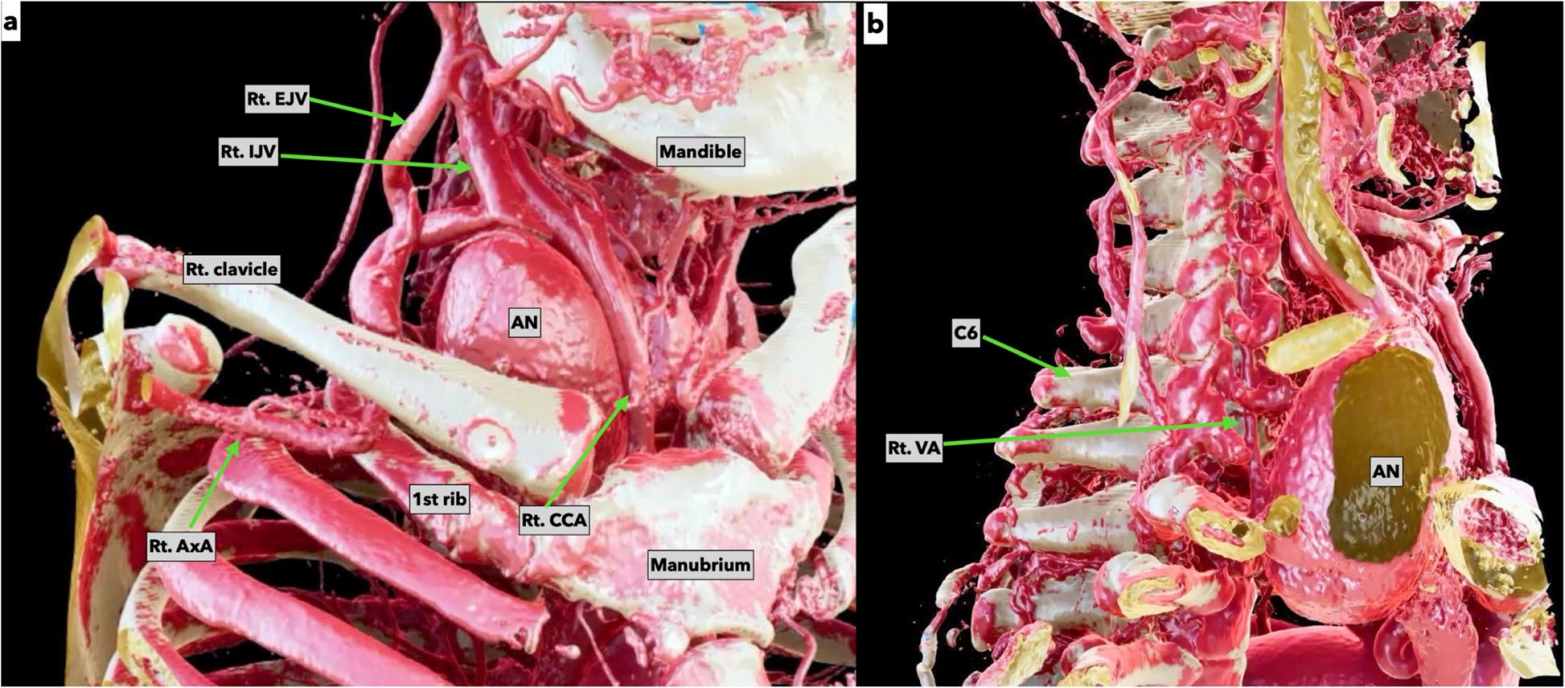
The anatomical orientation in case 1. **a** The orientation of the anatomical position of the right subclavian artery aneurysm from an anterior view is shown. This three-dimensional (3D) reconstruction image derived from the enhanced computed tomography was edited by Viewtify® technology, a software to instantaneously create 3D images. **b** The right side-view of the aneurysm is shown. The anatomy of the dorsal aneurysm is revealed. The positional relation between Rt. VA, C6, and the aneurysm become evident by Viewtify® technology. AN, aneurysm; Rt. CCA, right common carotid artery; Rt. EJV, right external jugular vein; Rt. IJV, right internal jugular vein; Rt.VA, right vertebral artery; C6, 6th cervical vertebrae; SCA, subclavian artery

**Fig. 3:**
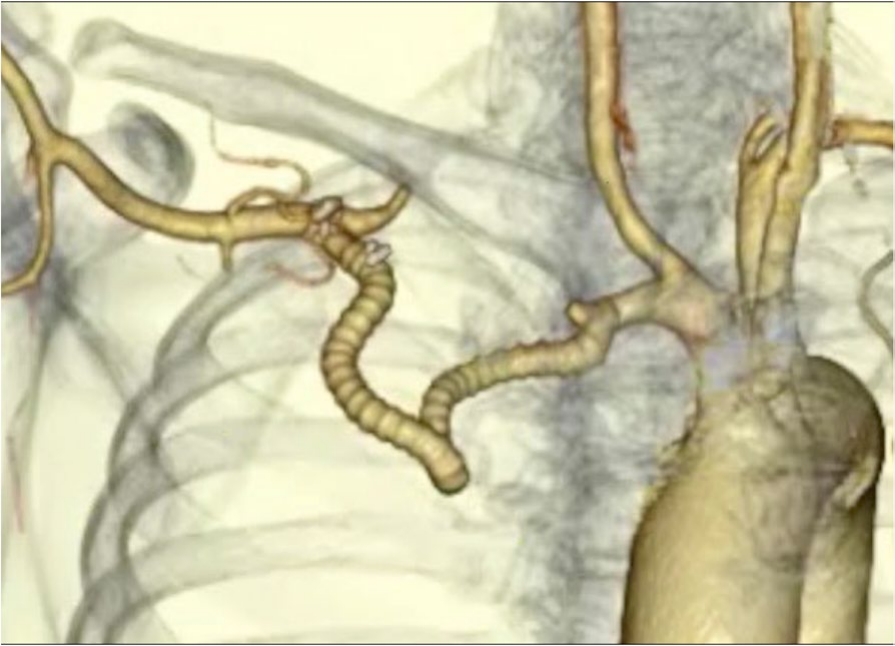
The 3D enhanced computed tomography image of the reconstruction of the right subclavian artery is shown

A 54-year-old man was referred to our hospital for a pulsatile mass in the right neck. He had no significant medical history. Contrast-enhanced computed tomography (CT) revealed a 50 × 80 mm true aneurysm in the right SCA compressing the airway and right lung. Because of the size of the aneurysm, it was deemed to be difficult to secure a working space even if a supraclavicular approach, infraclavicular approach, and median sternotomy were combined. The risk of massive bleeding from the branches originating from the aneurysm is another concern to secure a surgical view of the field through limited exposure (Fig. 2a, b). We were aware of the branches originating from the aneurysm and right vertebral artery before the surgery, but we refrained from performing preoperative embolization of those arteries to reduce intraoperative bleeding. We thought that manipulation of a catheter could induce multiple emboli of the thrombi attached to the aneurysmal wall. Preoperative magnetic resonance imaging (MRI) revealed no cerebrovascular circulation abnormalities. The left vertebral artery was dominant. Therefore, we applied TMA using an L-shaped incision from the medial side of the right sternocleidomastoid muscle to the right second intercostal space. Both the first and second rib cartilages were divided. The right axillary artery was simultaneously exposed via infraclavicular approach because we anticipated that the vessels might be located fairly deep via the TMA judging from the findings with preoperative computed tomography. Under femoro-femoral cardiopulmonary bypass (CPB) support, the orifices of the branches of the aneurysm were suture-closed and hemostasis was then achieved by use of cardiotomy suctions. It was difficult to dissect the right phrenic and vagus nerve adjacent to the aneurysm, because the enlargement of the aneurysm caused deviation of the nerves from its their normal locations and we could not detect the nerves in the surgical field. In an attempt to avoid damages to the nerves, we performed the minimal dissection around the aneurysm, not the entire aspects of the aneurysm. The aneurysmal wall was partially excised, and the distal and proximal SCA were suture-closed. The SCA was reconstructed using an ePTFE graft (GORE® PROPATEN® Vascular Graft 6 mm, GORE, Newark, DE, USA) from the right proximal SCA to the right axillary artery (Fig. 3). The CPB time was 53 min, and the operation time was 549 min. The postoperative course was uncomplicated, and the patient was discharged from the hospital without neurological complications on postoperative day 12. Three years after the surgery, no stenosis or pseudoaneurysm at the anastomotic site was observed.

### Case 2

**Fig. 4:**
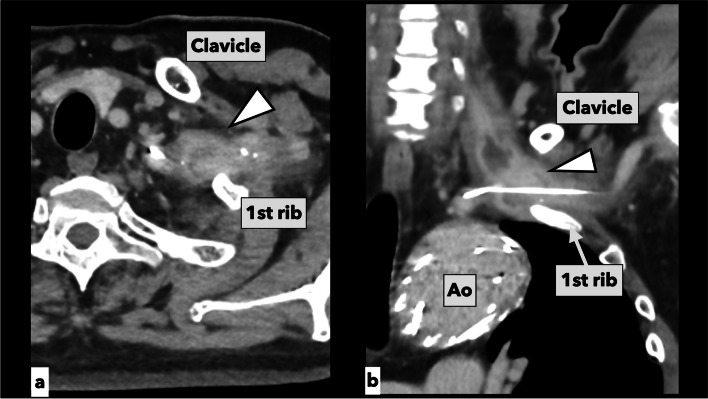
Enhanced computed tomographic image before antibiotics therapy in case 2. **a** Axial view of the abscess formation around the left subclavian artery and the guidewire penetrating the vessel wall of the left subclavian artery (white arrowhead). **B** Coronal view of the abscess formation (white arrowhead). Ao, aorta

**Fig. 5:**
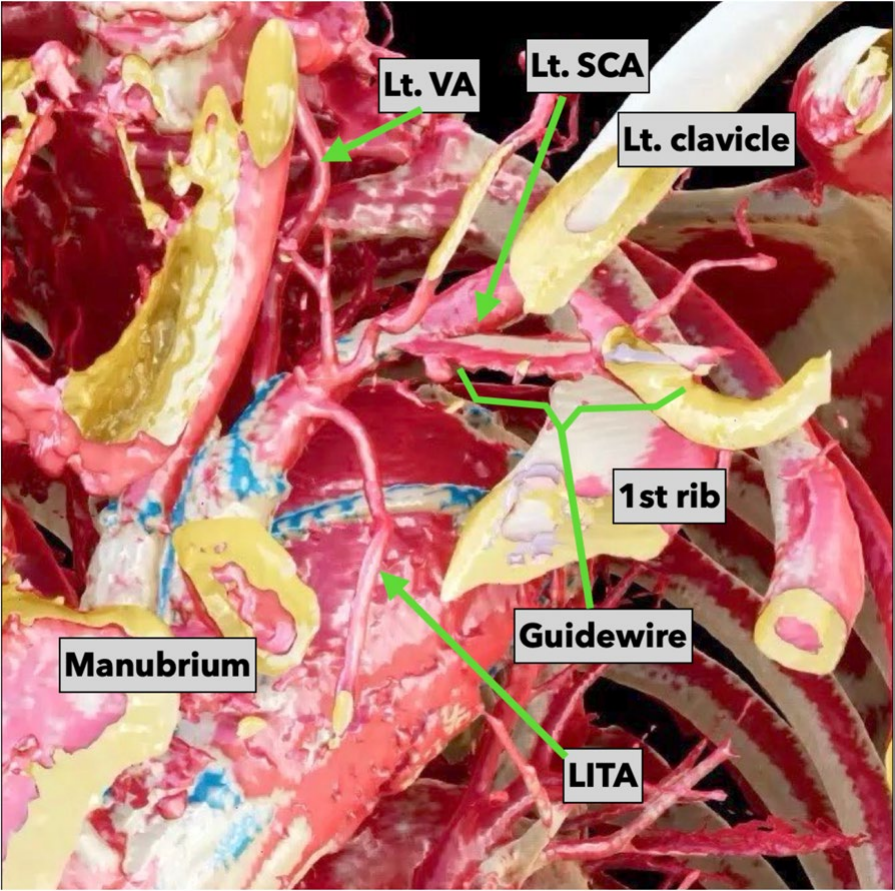
The three-dimensional computed tomographic angiography in case 2. The image shows an approach similar to the transmanubrial osteomuscular sparing approach. The point where the guidewire is penetrating the vessel wall is seen. Both figures are reconstructed from computed tomography data by utilizing Viewtify®. LITA, left interthoracic artery; Lt. SCA, left subclavian artery; Lt. VA, left vertebral artery

**Fig. 6:**
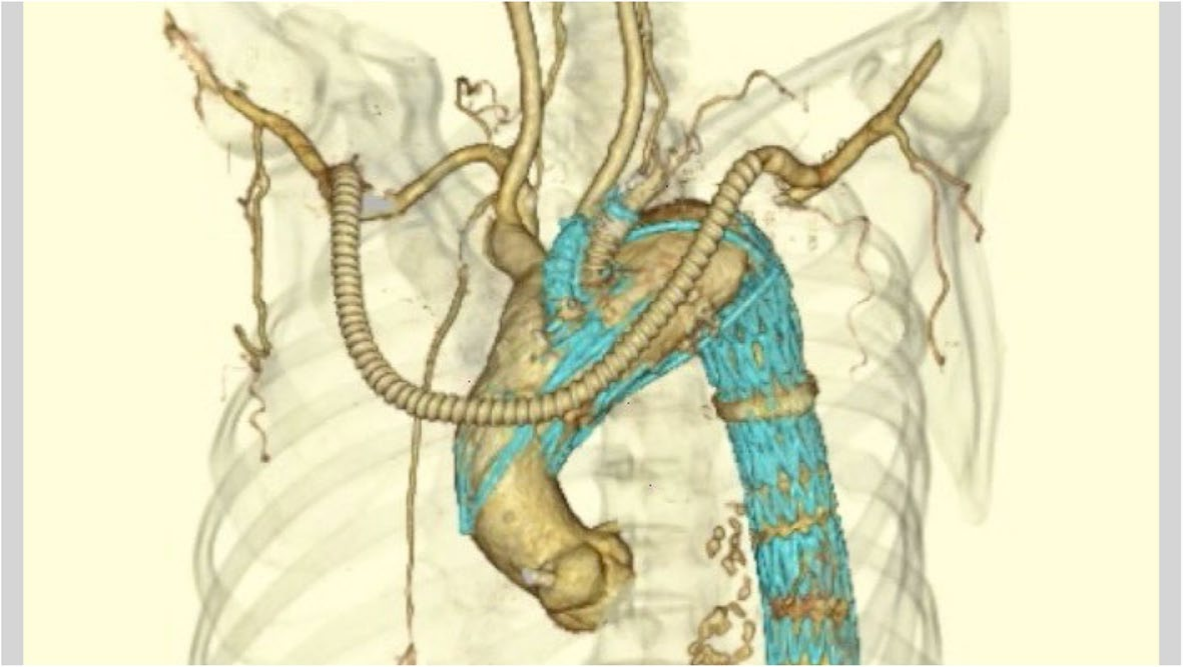
Postoperative three-dimensional computed tomographic angiography in case 2. Partial resection of the left subclavian artery and axillaro-axillary artery bypass was performed. Pseudoaneurysms were not detected around the stump of the left subclavian artery and left axillary arteries

A 72-year-old man underwent thoracic endovascular aortic repair (TEVAR) for the treatment of an aortic arch aneurysm at another hospital. During the surgery, the guidewire that passed through the left SCA was stuck and unable to be retrieved, and the wire was cut as short as possible and left inside the SCA. Four years later, abscess formation was detected by CT inspection around the left SCA and methicillin-susceptible *Staphylococcus aureus* bacteremia developed because of the guidewire (Fig. 4a, b). After administering antibiotics, we scheduled the removal of the remaining guidewire in the left SCA and the debridement of the infected lesion (Fig. 5). An L-shaped incision was placed from the medial aspect of the left sternocleidomastoid muscle to the first intercostal space, the first rib cartilage was divided, and the chest was opened using TMA under femoro-femoral CPB support in preparation for massive bleeding. We deemed that using cardiotomy suctions would reduce substantial loss of blood. Firstly, we performed an axillo-axillary bypass using an ePTFE graft (FUSION VASCULAR GRAFT 7 mm, MAQUET, Rastatt, Germany) (Fig. 6). Next, the left SCA was clamped distally at the site proximal to the bypass anastomosis, and also occluded proximally by an endovascular balloon (OPTIMO® EPD, external diameter: 9Fr, TOKAI MEDICAL PRODUCTS, Kasugai, Japan) at the portion proximal to the left vertebral artery origin. The severe adhesion surrounding the vessels required meticulous dissection of the left SCA under the ideal exposure. The guidewire was penetrated the vessel wall and trapped between the stentgraft and the aorta. A decision was made to remove the guidewire as much as possible and to isolate the left SCA. Following aggressive debridement of the infectious surrounding tissue, the left SCA was suture-closed between the portion distal to the left internal thoracic artery bifurcation and proximal to the axillo-axillary bypass anastomosis (Fig. 6). The CPB time was 512 min, and the operation time was 848 min. No postoperative complications occurred, and the patient was discharged home on postoperative day 12. Two years after the surgery, no recurrence of infection and no anastomotic abnormalities were observed.

### Case 3

**Fig. 7:**
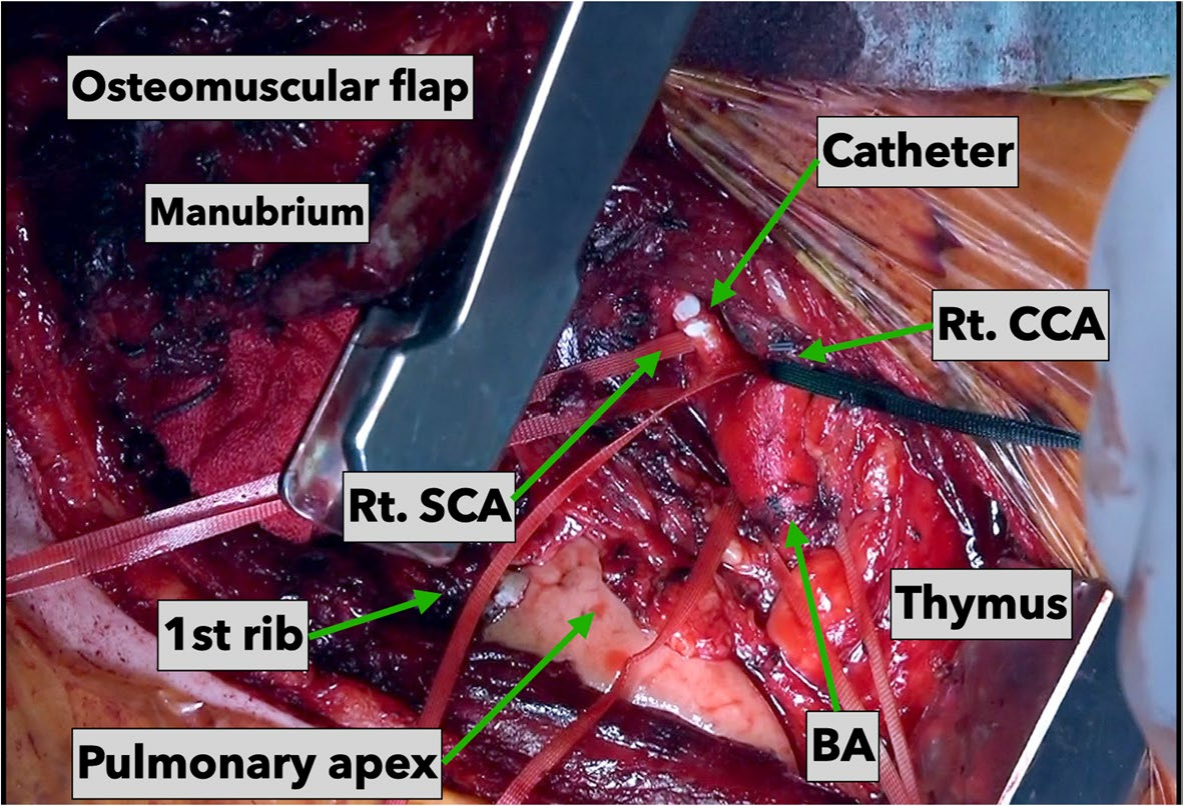
Operative findings using the transmanubrial osteomuscular approach in case 3. From the proximal brachiocephalic artery to the right subclavian artery, we could expose the vessels and secure working space enough to repair the vessels using the approach. BA, brachiocephalic artery; Rt. CCA, right common carotid artery; Rt. SCA, right subclavian artery

A 60-year-old man with renal failure and hyperkalemia was admitted to our emergency department. An indwelling dialysis catheter (External diameter: 12 Fr, Blood Access UK catheter kit, NIPRO, Osaka, Japan) was placed from the right neck into the right internal jugular vein for emergency dialysis. However, it was accidentally misplaced from the right SCA into the proximal aortic arch (Supplementary movie 1). With a prevision that manual external compression might be difficult to achieve while removing the catheter because of the anatomical location, we elected to perform an emergency surgery employing TMA. An L-shaped incision was made from the medial side of the right sternocleidomastoid toward the first intercostal space, and the first rib was resected. After exposing the brachiocephalic artery, right common carotid artery, and right SCA (Fig. 7), a purse-string suture was placed and the catheter removed (Supplementary movie 2). The operative time was 130 min. The surgery was completed without a cardiopulmonary bypass.

## Discussion and conclusions

A surgical approach for SCA could be concerned due to anatomical complexity. The presence of the clavicle and ribs often limits the working space and compromises the view of surgical field during open surgery. In the literature, the surgical approach to the right SCA is reportedly with supra- and/or infra-clavicular incision, and, if necessary, through a median sternotomy when the proximal SCA is involved. The approach to the left SCA is commonly a supraclavicular incision and/or left thoracotomy. In some cases, the clavicular resection is needed additionally [2]. Even in these combined approaches, the surgical view of the SCA could be still suboptimal. Furthermore, there is a risk of further injuries to surrounding structures of the SCA. Endovascular treatment could be another option for SCA aneurysms and injuries enabling a less invasive approach for SCA surgery [1–3]. However, its indications vary depending on the pathological lesion and anatomy, and the durability is questionable, given the wide range of motion of the shoulder and the potential for an intravascular device to be kinked by the first rib [3]. Additionally, there is a risk of endoleak in aneurysm cases due to branch vessels [2, 3, 7].

Since the publication by Grunenwald and Spaggiari in 1997 [6], TMA has been one of the common approaches for superior sulcus tumor surgery. TMA was firstly applied for a SCA surgery and reported by Kim et al. in 1998, in which a true 45 × 25 mm aneurysm was replaced with an artificial graft [8]. To our best knowledge, this Kim’s publication is the first and the only report regarding the application of TMA in vascular surgery. Taking advantage of the property to overcome the inherent anatomical difficulties regardless of patient’s settings, we have expanded the application of TMA to various types of SCA surgeries. The case 1 was a young patient with a large SCA aneurysm that was compressing the trachea and right lung. TMA with partial sternotomy was useful to remove the sizable aneurysm and replace with the prosthetic graft; otherwise, the surgical view would have been quite limited. Case 2 involved an infection of the SCA that required debridement. An ideal view of the surgical field was necessary because the degree of adhesion was severe and thorough debridement of all infectious tissue was required. Case 3 was a case of iatrogenic trauma. TMA was the only way to secure our hemostasis and complete the surgery because of the critical anatomy, i.e., an area around the common carotid artery and SCA bifurcation from the brachiocephalic artery, that is rarely approached. Similarly, the use of TMA has been reported for the repair of perforation into the confluence innominate vein and right jugular vein due to the misplacement of an indwelling dialysis catheter [9]. Our present report is the first case of applying a TMA to remove a catheter from the artery. The trap-door approach also allows for wide exposure of the subclavian artery, but differs in that it does not preserve the sternocleidomastoid muscle and limits the approach to the common carotid artery.

Regarding the invasiveness of the surgery, although TMA necessitates an excision of the superoexternal part of the manubrium, this does not cause any functional and aesthetic disadvantages. Because TMA leaves in situ the clavicle sparing major part of the pectoral muscle, shoulder girdle movement is fully preserved postoperatively. The size of the skin incision is relatively big compared to other open surgical approaches, and the rate of wound complication was reportedly low [6, 8, 10]. These functional and cosmetic advantages of TMA enable us to apply this approach even in pediatric cases. Madi et al. described that no patients suffered from postoperative chest deformity and the only long-term morbidity observed in a few patients was a discrete atrophy of the pectoralis muscle [10].

To conclude, the TMA is not only a simple and safe procedure, but also a highly useful approach for variable SCA surgeries, given the wide range of applications in various pathological and clinical settings. This approach provides an excellent surgical field of the entire subclavian artery, preserves the shoulder function, and prevents wound complication even in an emergency case. Open repair of the SCA is still a primary procedure in majority of cases requiring SCA reparative procedures, and TMA can be the standard surgical approach for those cases.

## Supplementary Information

Supplementary Movie 1. The movie shows a three-dimensional image of the misplacement from the right subclavian artery into the proximal aortic arch derived from enhanced computed tomography in case 3, edited by Viewtify® technology.

Supplementary Movie 2. The movie is the intraoperative video clip of case 3. TMA is applied to remove an indwelling dialysis catheter from the right subclavian artery.

## Data Availability

The data sets supporting the conclusions of this study are included within the article and its additional files.

## Abbreviations

CPB: Cardiopulmonary bypass
CT: Computed tomography
ePTFE: Expanded polytetrafluoroethylene
MRI: Magnetic resonance imaging
SCA: Subclavian artery
TEVAR: Thoracic endovascular aortic repair
TMA: Transmanubrial osteomuscular sparing approach
